# Supplementary material for: SERPINF1 Mediates Tumor Progression and Stemness in Glioma
Source: Genes (Basel). 2023 Feb 25;14(3):580. doi: 10.3390/genes14030580 (PMC10047918; doi:10.3390/genes14030580)

**Table S1.** The sequences of siRNAs and primers.

| Name | Sequence |
|---|---|
| si-SERPINF1-1 | 5'-CCCGGATCGTCTTTGAGAA-3' |
| si-SERPINF1-2 | 5'-GAACAGAATCCATCATTCA-3' |
| SERPINF1 forwar( | 5'-TGTCTCCAACTTCGGCTATG-3' |
| SERPINF1 reverse | 5'-AGTAGAGAGCCCGGTGAATG-3' |
| GAPDH forward | 5'-ACCCACTCCTCCACCTTTGAC-3' |
| GAPDH reverse | 5'-TGTTGCTGTAGCCAAATTCGTT-3' |

**Supplementary Figure S1.** Knockdown efficiency of SERPINF1 in A172 and LN18 verified by Western blot.

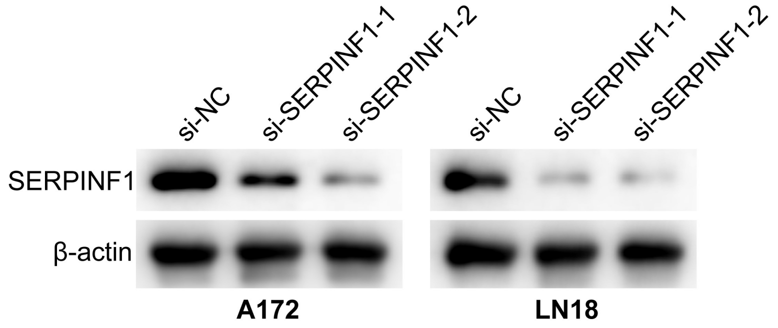


**Supplementary Figure S2.** Cellular morphological images of A172 and LN18 after SERPINF1 knockdown.

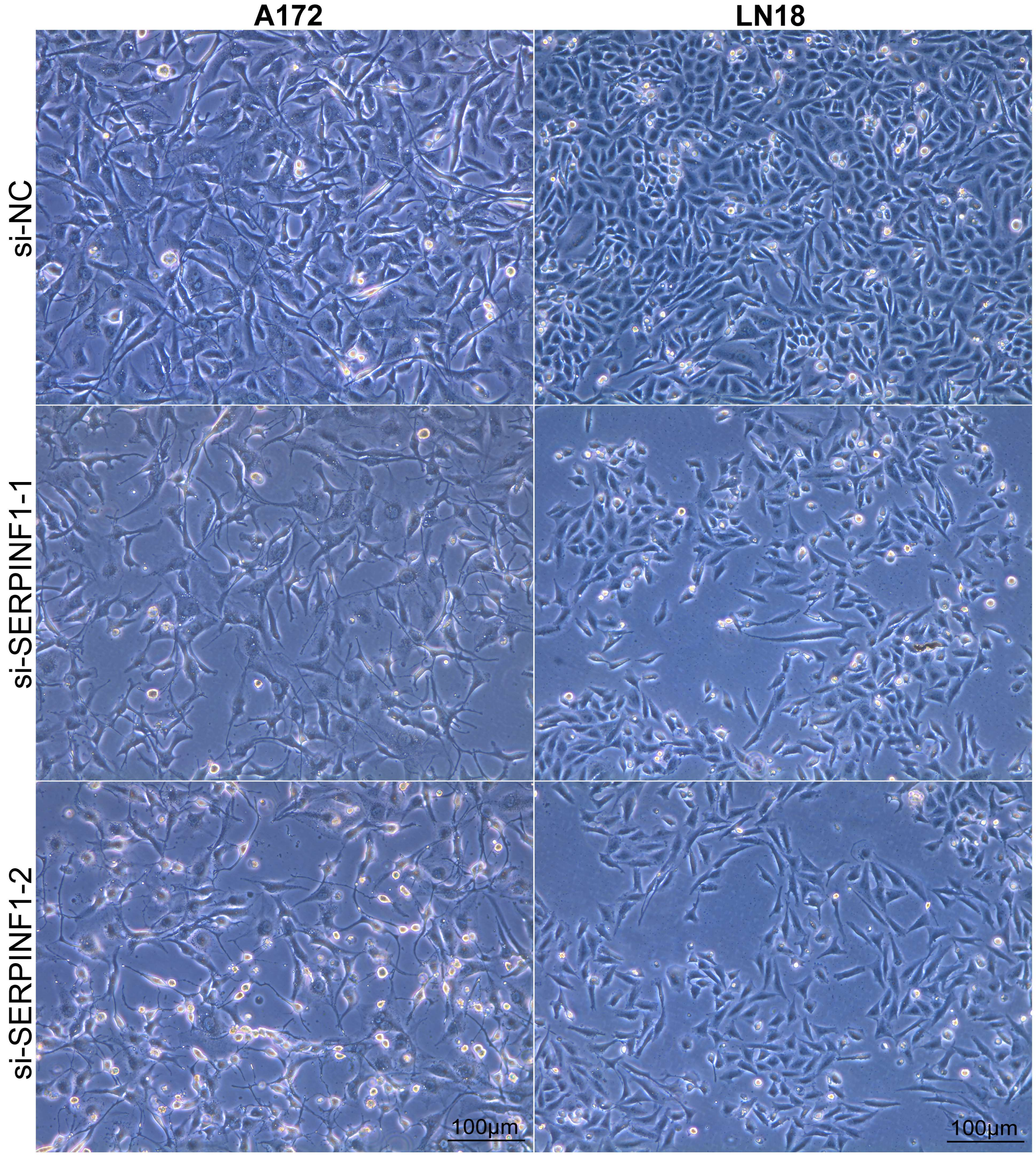

**Supplementary Figure S3.** ChIP-seq data of the TFs in Cistrome Data Browser revealed that STAT1, CREM, and NR2F2 were highly enriched in the promoter region of SERPINF1 in K562, HepG2, and SET2 cells. (See https://tiny.one/ycksjbwd for detail.)

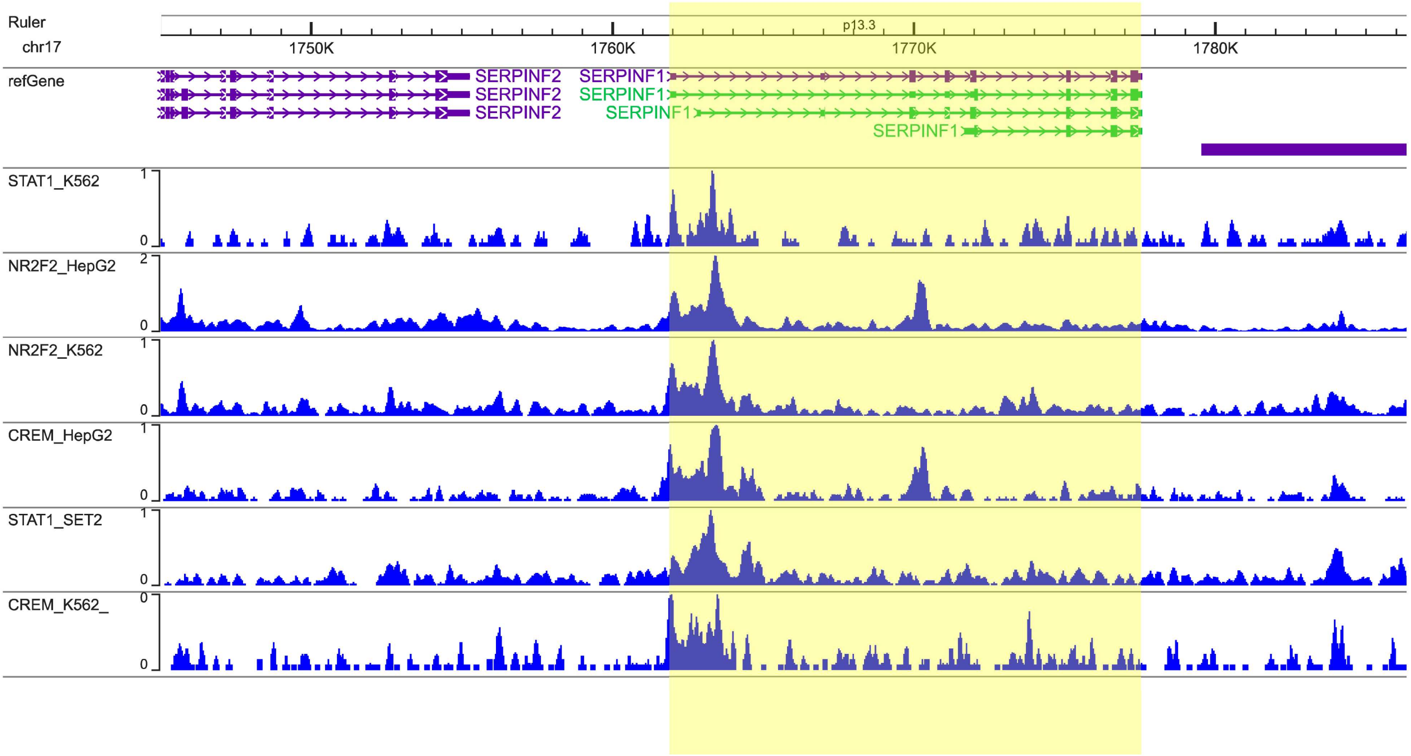

Supplement: Supplementary file 1 [file genes-14-00580-s001.zip › genes-2212042-supplementary.pdf]
